# Mid-term outcomes after open-heart surgery for severe chronic rheumatic heart disease in a low-income country: an observational study with historical controls

**DOI:** 10.3389/fcvm.2026.1763889

**Published:** 2026-03-11

**Authors:** Stale Wagen Hauge, Mette E. Estensen, Robert Matongo Persson, Dejuma Yadeta, Chala Fekadu Oljira, Abebe Tessema, Vegard Ellensen, Atle Solholm, Thomas Dolven, Nigussie Bogale, Hans Martin Flade, Kjell Vikenes, Havard Dalen, Rune Haaverstad

**Affiliations:** 1 Department of Heart Disease, Haukeland University Hospital, Bergen, Norway; 2 Department of Circulation and Medical Imaging, Norwegian University of Science and Technology, Trondheim, Norway; 3 Clinic of Cardiology, St. Olavs University Hospital, Trondheim, Norway; 4 Department of Cardiology, Oslo University Hospital, Oslo, Norway; 5 Department of Clinical Science, Faculty of Medicine, University of Bergen, Bergen, Norway; 6 Department of Cardiology, School of Medicine, CHS, Addis Ababa University, Addis Ababa, Ethiopia; 7 Department of Surgery, School of Medicine, CHS, Addis Ababa University, Addis Ababa, Ethiopia; 8 Department of Surgical Services, Haukeland University Hospital, Bergen, Norway; 9 Department of Surgical Services, St. Olavs University Hospital, Trondheim, Norway; 10 Department of Medicine, Levanger Hospital, Levanger, Norway

**Keywords:** echocardiography, heart valve prosthesis, heart valve surgery, mitral regurgitation, mitral stenosis, rheumatic heart disease, sub-Saharan Africa

## Abstract

**Objectives:**

The literature is sparse regarding outcomes after valvular surgery in patients with severe chronic rheumatic heart disease (RHD) in low- and middle-income countries (LMICs). We aimed to evaluate mid-term outcomes in patients with severe chronic RHD who underwent open-heart surgery in Ethiopia, and evaluate the durability of the operated valves during follow-up.

**Methods:**

This study implemented an observational design, with 104 patients screened for cardiac surgery, of whom 52 were excluded. Outcome measures and clinical and echocardiographic data after cardiac surgery were available for the remaining 52 patients. Survival was compared with that of a cohort of 157 control patients recruited from the waiting list based on similar characteristics. Of these patients, 108 were lost to follow-up or underwent surgery or interventions performed by other missions, leaving 49 for comparison. The functioning of the repaired valves or valvular prosthesis was examined by echocardiography.

**Results:**

The mean follow-up time among the 52 (65% women) surgical patients and 49 (76% women) controls was 4.1 years and 3.6 years, respectively. In the surgical group, 46 (88%), 25 (48%), and 18 (35%) patients underwent mitral, tricuspid, and aortic valve surgeries, respectively. The survival rate at last follow-up was 83% in the surgical group and 57% in the control group (*P* = 0.004). At their last follow-up, four patients had moderate valvular obstruction and none had severe valvular dysfunction.

**Conclusions:**

Cardiac surgery for severe chronic RHD is feasible in LMICs if the service is well-structured and can improve survival at mid-term follow-up. The durability of both repaired and replaced valves was good.

## Introduction

Rheumatic heart disease (RHD) is a major cause of morbidity and mortality worldwide and constitutes a significant burden to healthcare systems in low- and middle-income countries (LMICs) (1). More than 30 million patients are affected by acute rheumatic fever and subsequent RHD globally and RHD accounts for more than 300,000 deaths annually (2). Surgery is the only curative treatment when severe disease is present (3–5) and is a well-established treatment in high-income countries (6–10). However, surgical treatment for RHD in LMICs is less documented. While short-term follow-up data after surgery for RHD have shown acceptable results (1, 11–17), studies with longer follow-up time are lacking, especially for surgical valve replacement (17–21). The natural course of severe RHD treated medically is also uncertain (11, 22–25).

Ethiopia, according to the UN's Human Development Index (www://data.undp.org/countries-and-territories/ETH) for 2023, is in the low human development category, as it is ranked 180 out of 193 countries and territories. Recently, a large African registry [Global Rheumatic Heart Disease Registry (REMEDY)] has highlighted the high mortality and morbidity associated with RHD, and a recent report demonstrated that, despite efforts to make cardiac surgery service available across Africa, the progress has been slow, rendering RHD fatal for most patients (26). In Africa, there is one cardiac surgery center per 33 million people, with a ratio of almost 1:300 compared to the United States (one center per 120,000 people) (26). Moreover, no private or governmental centers offer regular cardiac surgery services in Ethiopia. These facts constituted the rationale for a collaborative mission where a Norwegian heart team regularly visited Ethiopia to build sustainable cardiac surgery services at Tikur Anbessa Specialized Hospital, Addis Ababa, Ethiopia. Using data from these missions, we aimed to evaluate mid-term survival after cardiac surgery for RHD and compare the outcomes with control patients with similar disease phenotypes who were not offered surgery or intervention due to limited capacity. A second aim was to study the durability of valvular prostheses and repaired valves after surgery for severe chronic RHD. The main hypothesis was that the operated patients would have better mid-term survival compared to the conservatively treated group, and that the patency of valve prosthesis and repaired valves was acceptable at follow-up despite the challenging environment.

## Methods

Comprehensive summaries of the study methodology have recently been published (1, 27).

### Study design and population

This was an observational study that included the biannual follow-up visits of operated patients. The outcomes of the surgically treated patients were comprehensively assessed and compared with medically treated patients with RHD with similar valvular disease severities who were not offered percutaneous intervention or cardiac surgery due to the limited capacities of the healthcare providers.

**Figure 1:**
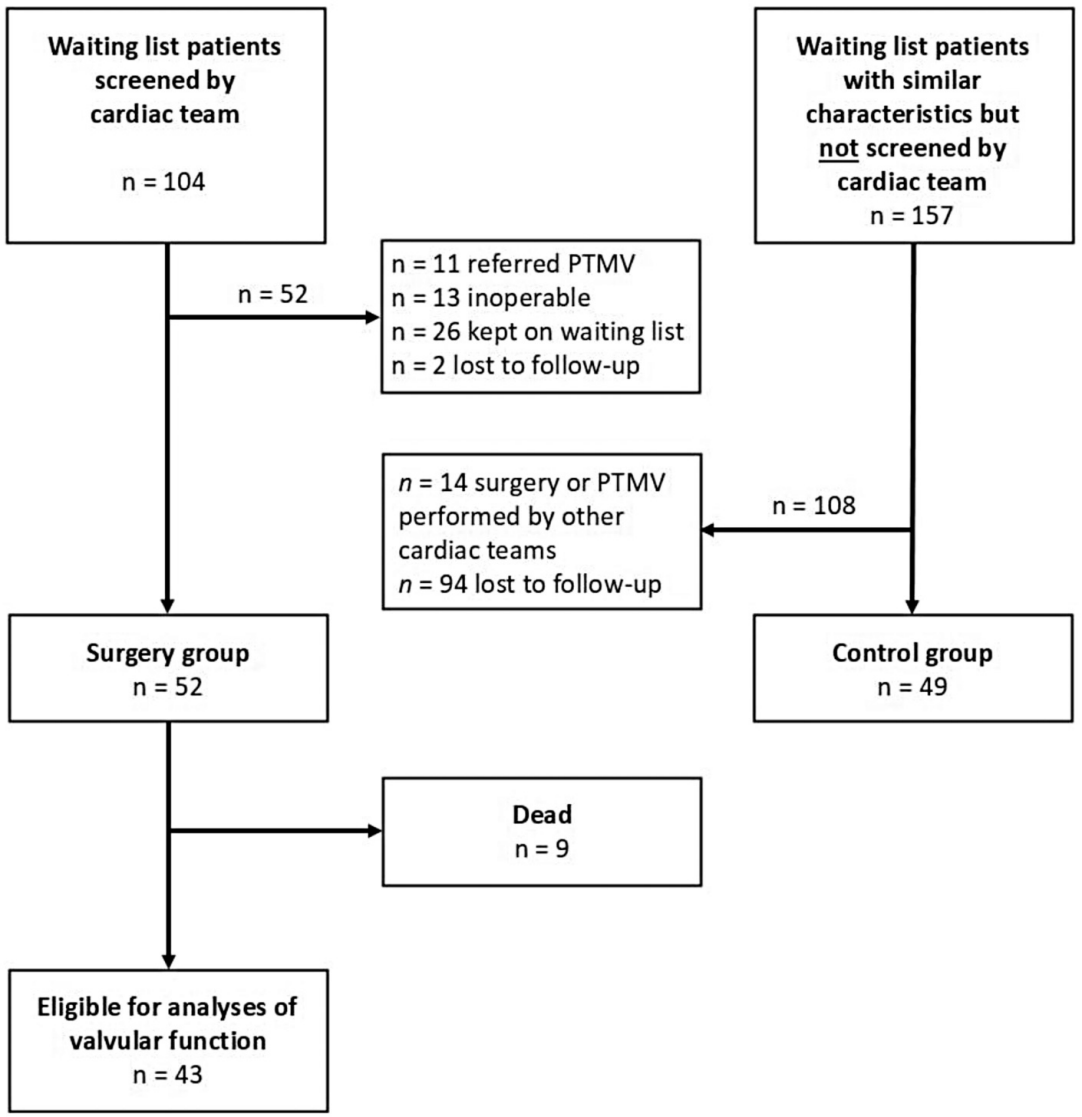
Flow chart of the study population.

Figure 1 shows the study flow chart. From the local RHD waiting list for open-heart surgery, consisting of approximately 6,000 patients, the administrative medical staff selected 104 for surgical screening by the Norwegian heart team during six missions. The criteria to select patients for surgical screening were as follows: (1) compliant with follow-up, (2) eligible for intervention, and (3) longest time on waiting list for surgery. Of the 104 patients screened between March 2016 and May 2023, we excluded 52, as 11 were referred for percutaneous mitral valvulotomy, 26 were kept on the waiting list, 13 were inoperable, and two were lost to follow-up. Thus, 52 operated patients were included in the surgical group for the analyses. Decisions on how to treat the screened patients were made by the heart team based on comorbidity, valvular disease phenotype and severity, and compliance with anticoagulation therapy. The Norwegian heart team had no relevant limitations with respect to the type of valvular surgery that could be offered, and the decisions on whether to replace or repair the diseased valves were based on clinical information only. To generate a historical control group, 200 patients were randomly selected from the waiting list for open heart surgery in November 2019. Information on personal identification, age, sex, echocardiographic details, and the time of surgical referral was available from the waiting list. The randomly selected 200 patients were balanced to the 37 RHD patients who had undergone surgery by the Norwegian team at that time point. Balancing was performed with respect to the distribution of the operated patients' age when placed on the waiting list, sex, number of diseased valves, pulmonary artery pressures, and time on the waiting list. Of the randomly selected patients, 43 were not possible to identify, leaving 157 patients in the control group. Of these, 94 were lost to follow-up, and 14 underwent surgery or percutaneous mitral valvulotomy performed by other missions. Thus, 49 control group patients were included.

Echocardiographic data from the surgical patients’ last follow-up visits were scrutinized to assess the function of the operated valves. The reproducibility of echocardiographic measurements and valvular functional classifications was evaluated in 15 randomly selected patients using data from the last follow-up visit. For the latter, two experienced echocardiographers (SH and HD) performed analyses that were blinded to each other. Data from the reproducibility study are presented in the Supplementary Material.

The study was approved by the Regional Committee for Health Research Ethics in Norway (ID 7179) and by the local ethics committee for medical research at Addis Ababa University in Ethiopia. The study was conducted in agreement with the second Declaration of Helsinki.

### Data collection

Data were collected by the heart team at baseline and by local and Norwegian health professionals throughout the follow-up period. A physical examination, including vital signs, anthropometric measurements, a 12-lead ECG, and blood samples, was part of the screening and follow-up examinations. Blood samples drawn at screening were analyzed in the Tikur Anbessa Specialized Hospital's laboratory. A hand-held CoaguCheck Pro II (Roche Diagnostics, Basel, Switzerland) was used to monitor the effect of anticoagulants using the International Normalized Ratio (INR) in the surgical patients. The use of antibiotics, anticoagulants, diuretics, beta-blockers, angiotensin-converting enzyme (ACE) inhibitors, and digoxin was recorded. We defined heart failure by the following criteria: New York Heart Association (NYHA) class ≥ II, structural heart disease, and at least one of the following: (1) at least two heart-failure medications (furosemide, spironolactone, beta-blocker, ACE-inhibitor, and digoxin); (2) left ventricular (LV) ejection fraction <50%; (3) peak tricuspid regurgitant velocity ≥3 m/s; (4) pleural effusion or ascites; and (5) history of hospitalization for heart failure. Heart failure symptoms were classified according to the NYHA guidelines (28). The EuroSCORE II calculator was used to assess the risk for the surgical patients (29).

### Echocardiography

Cardiologists from the Norwegian heart team and a local cardiologist performed all the echocardiographic examinations in the surgical group. All the analyses were performed by one experienced cardiologist echocardiographer (HD). In the control group, all the echocardiographic evaluations were performed by local cardiologists. LV volume and ejection fraction were calculated from the apical four-chamber (A4C) and two-chamber (A2C) views using Simpson's method. LV stroke volume and cardiac output were calculated using Doppler measurements and indexed for body surface area. Systolic pulmonary artery pressure was estimated using the peak tricuspid regurgitation gradient on continuous Doppler in A4C, plus the right atrial pressure estimated from the size and respiratory variation of the inferior vena cava (30). The Wilkins score was used as a severity marker of rheumatic mitral valves (31). Mitral stenosis (MS) severity was graded based on the calculated valve area using planimetry in parasternal short-axis views and/or by the mitral inflow Doppler spectrum using the pressure half-time method (area = 220/pressure half-time) (32). Mitral regurgitation grading was based on valvular morphology, the regurgitant jet color flow spectrum, and semiquantitative measurements, such as vena contracta and signs of systolic pulmonary vein flow reversal. In the surgical group, mitral regurgitations were also evaluated using the proximal isovelocity hemispheric surface area (PISA) method. The severity of all valvular stenoses or regurgitations was evaluated according to current recommendations (33, 34). The same approach was used for follow-up echocardiograms, including the assessment of repaired valves and implanted prostheses. The choice of which cycles or recordings were best suited for each of the analyses was left to the operator. All echocardiographic recordings were acquired using a GE Vivid E9 or a Vivid *i* ultrasound scanner (GE HealthCare, Horten, Norway). All the echocardiographic analyses of the surgical patients presented here were conducted offline using EchoPAC SWO (versions 201–206, GE HealthCare).

### Surgery, follow-up, and outcomes

Open-heart surgery was performed with median sternotomy using general anesthesia and cardiopulmonary bypass circulation. Recommendations from the heart team were discussed with each patient in a shared decision process before the operative treatment. The choice of valvular prosthesis was based on valve pathology, the patient’s age, compliance with anticoagulation treatment, comorbidity, family planning, and rural housing issues. Postoperative pacemaker implantation was performed when clinically indicated. Anticoagulation treatment with oral warfarin was provided, aligning with international guidelines for patients with mechanical valves or other indications (33). The target anticoagulation level was INR 2.5 (range 2.0–3.0) in cases with a mechanical aortic valve only and INR 3.0 (range 2.5–3.5) for cases with a mechanical valve in the mitral position. Additional aspirin was not routinely used. Clinical evaluation, 12-lead ECG, transthoracic echocardiography, and laboratory testing were performed before discharge and at follow-up visits scheduled every 6 months. If cardiac or prosthetic heart valve thrombosis was suspected by transthoracic echocardiography, a supplementary transesophageal examination and fluoroscopy were used to confirm the diagnosis and optimized warfarin treatment was given special attention.

Perioperative and 1-year outcomes have been detailed recently (1). Surgical patients were regularly assessed at each follow-up visit using patient interviews and by scrutinizing the medical record files. The control group was assessed using medical record files, outpatient visits, and telephone calls. The primary outcome measure was mortality. In cases where death could not be confirmed by medical records or a telephone interview with family members, the patients were classified as lost to follow-up. We used the recommendations from the European Association of Cardiovascular Imaging (35) to define outcome measures related to prosthetic valves, while repaired valves were classified on the discretion of the experienced cardiologist echocardiographer (HD) based on valve morphology, Doppler measurements of blood flow through the valve and adjacent orifices, the direction and distribution of the color Doppler spectrum, semiquantitative measurements (vena contracta width), and quantitative measurements (PISA). Prosthetic valves were classified as dysfunctional if any of the following were present: significant valve obstruction, severe regurgitation, or severe paravalvular leakage. The repaired valves were classified as dysfunctional if more than moderate regurgitation and/or stenosis was present.

### Statistical analyses

Continuous variables are presented as mean and standard deviation (SD) or as median and interquartile range (IQR), depending on their distribution. Normality was evaluated using histograms and Q-Q plots. Frequencies and proportions are used to present categorical variables. Student’s *t*-test and the Wilcoxon test were used for comparisons between the groups as appropriate. Proportions were compared using the chi-square test and Fisher’s exact test. Survival analyses were performed using the Kaplan–Meier method with the log-rank test to ascertain the difference between the groups. The variable used for follow-up time differed between the groups as follows: time from surgery in the surgical group and time since being placed on the waiting list for surgery in the controls. No missing data were imputed.

In cases without any event, operated patients were censored at their last follow-up visit, and controls were censored at their last communication with the local doctors, either by telephone or physical visit to the outpatient clinic. *P*-values <0.05 were considered statistically significant. All statistical analyses were performed using IBM SPSS Statistics 28 (IBM Corp., Armonk, NY, USA).

## Results

### Population

Baseline characteristics indicating the chronic severe state of the study population are shown in Table 1. The patients in the surgical group had a mean age of 30 years, approximately 4 years older than the controls (*P* = 0.02). There were more women than men in both groups (65% in the surgical group and 76% in the controls, *P* = 0.29). At baseline, the patients in the surgical group had been symptomatic for a median of 11 years compared to 3 years among the controls (*P* < 0.01). The patients in the surgical group had several characteristics indicating more advanced disease than the controls, such as a higher burden of atrial fibrillation (*P* < 0.01), more thromboembolic events (*P* = 0.03), more anticoagulation therapy (*P* < 0.01), and more beta-blocker therapy (*P* = 0.03).

**Table 1:** Basic characteristics of the study population.

Variable	Surgical group	Control group	*P*-value
Number	52	49	
Women, *n* (%)	34 (65)	37 (76)	0.32
Age, mean (SD), years	29.9 (9.3)	25.9 (9.9)	0.02
NYHA class III or IV, n/a (%)	27/52 (52)	21/37 (57)	0.53
Height, mean (SD), cm	162 (9.1)	–	
Weight, mean (SD), kg	56.4 (13.2)	–	
Systolic blood pressure, mean (SD), mm Hg	117 (21)	107 (15)	<0.05
Diastolic blood pressure, mean (SD), mm Hg	71 (11)	66 (12)	<0.05
Heart rate, median (IQR), beats per minute	80 (74–89)	80 (78–92)	0.28
Serum creatinine, mean (SD), mg/dL	0.87 (0.26)	–	
Time with symptoms, median (IQR), years^a^	11 (7–15)	3 (1–5)	<0.01
Use of antibiotics, n/a (%)	42/52 (81)	–	
Use of anticoagulation, n/a (%)	28/52 (54)	6/42 (14)	<0.01
Use of beta-blocker, n/a (%)	20/52 (39)	6/49 (12)	0.03
Use of diuretics, n/a (%)	39/52 (75)	34/48 (71)	0.66
Euroscore II (%)	2.6 (1.7)	–	
Complications of RHD			
Atrial fibrillation, n/a (%)	24/52 (46)	7/39 (18)	<0.01
Thromboembolic disease, n/a (%)^b^	6/52 (12)	0/41 (0)	0.03
Stroke, n/a (%)	2/52 (64)	0/41 (0)	0.5
Infective endocarditis, n/a (%)	3/52 (6)	2/41 (5)	1.0
Heart failure, n/a (%)^c^	42/52 (81)	36/49 (74)	0.48

a For the surgical patients, years with symptoms before surgery, and for the controls, years with symptoms before being placed on the waiting list.

b Thromboembolic disease: peripheral emboly, left atrial thrombus, and valvular thrombus.

c Heart failure: NYHA ≥2, structural heart disease, and at least one of the following: more than two heart failure medicines, EF <50%, tricuspid regurgitation maximal velocity >3 m/s, pleural effusion or ascites, or heart failure hospitalization.

### Echocardiography

**Figure 2:**
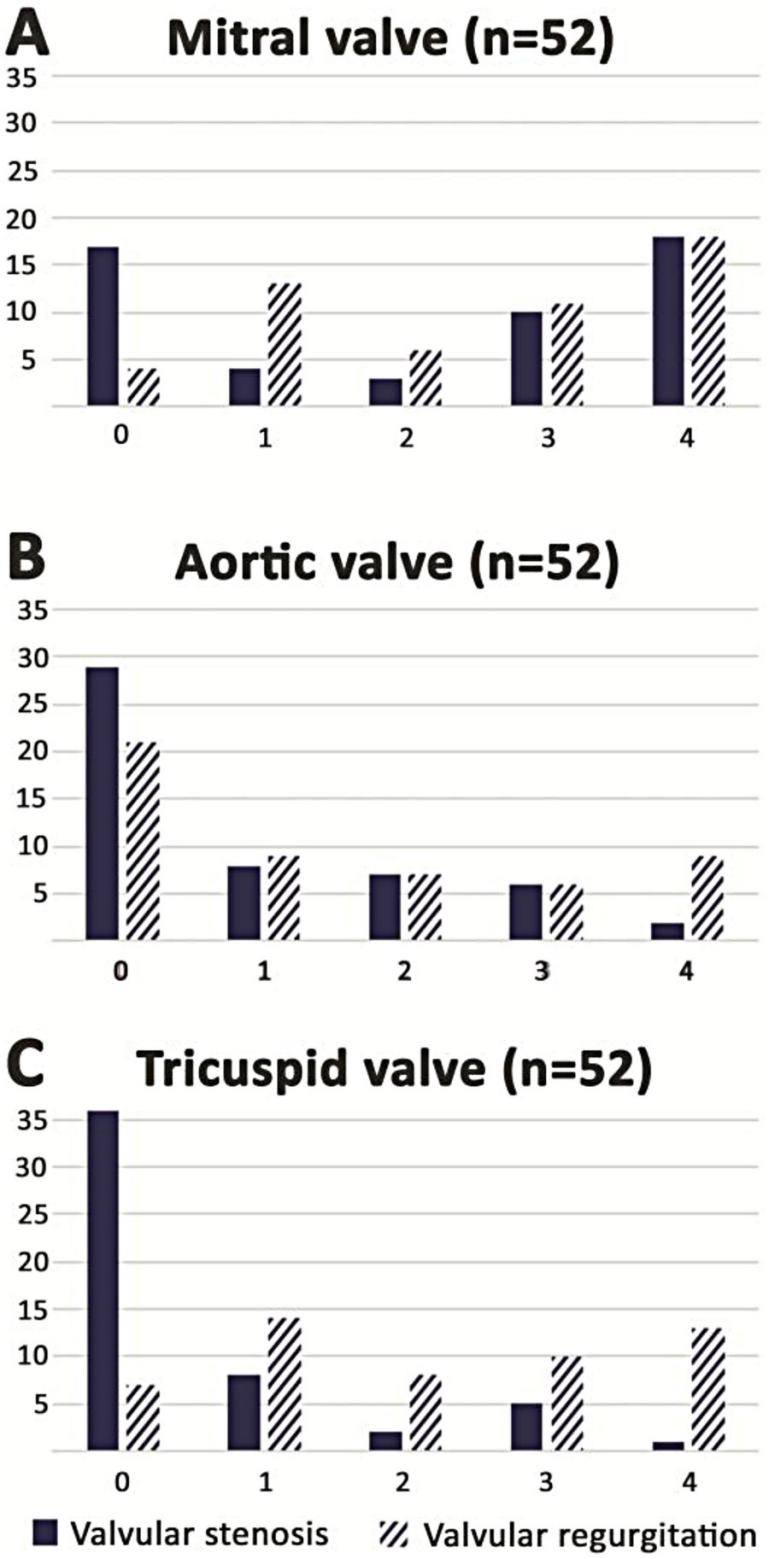
Valvular pathology during the screening of the 52 operated patients. (0 = none, 1 = mild, 2 = mild to moderate, 3 = moderate, and 4 = severe). Trace (0.5) is included in group 1 (mild). None of the patients had > mild pulmonic valve disease. **(A)** Mitral valve. **(B)** Aortic valve. **(C)** Tricuspid valve.

Preoperative echocardiographic data are summarized in Table 2. The majority of the patients had moderate or severe pathology in several valves, with no significant differences between the groups (*P* = 0.06). Mitral valve disease was the most frequent pathology among the surgical patients, whereof 54% had at least moderate stenosis and 56% had at least moderate regurgitation, whereas stenosis was more common than regurgitation in the controls. Tricuspid regurgitation was the second most common valve disease in both groups. Figure 2 illustrates the detailed grading of the mitral, aortic, and tricuspid disease in the surgical patients at screening. Indexed LV stroke volume was low (median of 31 mL/m^2^) among the surgical patients, even though LV ejection fraction was in the normal range (mean of 56%). The majority of the surgical patients had pulmonary hypertension and their mean systolic pulmonary artery pressure was 49 mmHg.

**Table 2:** Baseline echocardiographic data.

Variable	Surgical group	Control group	*P*-value
Number	52	49	
Left ventricular ejection fraction ≤50%, n/a (%)	15/52 (29)	5/36 (14)	0.13
SPAP ≥60 mmHg, n/a (%)	17/52 (33)	9/14 (64)	0.06
Number of valves affected, mean (SD)	1.8 (0.68)	1.5 (0.87)	0.06
Mitral stenosis (≥moderate), n/a (%)	28/52 (54)	34/42 (81)	0.01
Mitral regurgitation (≥moderate), n/a (%)	29/52 (56)	17/42 (41)	0.15
Aortic stenosis (≥moderate), n/a (%)	8/52 (15)	3/42 (7)	0.3
Aortic regurgitation (≥moderate), n/a (%)	15/52 (29)	14/42 (33)	0.66
Tricuspid stenosis (≥moderate), n/a (%)	6/52 (12)	3/42 (7)	0.72
Tricuspid regurgitation (≥moderate), n/a (%)	23/52 (44)	14/42 (33)	0.3
Pulmonary stenosis (≥moderate), n/a (%)	0/52 (0)	1/42 (2)	0.45
Pulmonary regurgitation (≥moderate), n/a (%)	0/52 (0)	0/42 (0)	1
Left ventricular end-diastolic diameter, mean (SD), mL	53 (10)	–	–
Left ventricular end-diastolic volume, mean (SD), mL	129 (69)	–	–
Left ventricular ejection fraction, mean (SD), %	56 (9)	–	–
Tricuspid annular plane systolic excursion, mean (SD), mm	20 (5)	–	–
Indexed left atrial volume, median (IQR), mL/m^2^	99 (71–131)	–	–
Indexed left ventricular stroke volume, median (IQR), mL/m^2^	31 (22–50)	–	–
Systolic pulmonary arterial pressure, mean (SD), mm Hg	49 (21)	–	–
Wilkins score, mean (SD)	10.4 (2.4)	–	–

Detailed echocardiographic data were only available in the surgical group.

### Patient outcomes

Comprehensive details of the surgeries and the early outcomes have recently been described (1). Some of these data are summarized in Table 3. Mitral valve surgery was most common, performed in 46 patients (88%) and mechanical valve implantation was the most frequently performed procedure in the mitral position. Tricuspid valve repair was the most common valvular repair, performed in 23 patients (44%). Surgical repair procedures included annuloplasty, commissurotomy, leaflet extension, and sub-valvular procedures.

**Table 3:** Data from the postoperative echocardiography of the 52 operated patients.

Variable	n/a (%)	Stenosis/obstruction (≥moderate)	Regurgitation (≥moderate)
Total mitral valve surgery	39/46 (85)		
Mitral valve repair	8/8 (100)	0 (0)	0 (0)
Mechanical prosthesis	30/37 (81)	0 (0)	–
Biological prosthesis	1/1 (100)	0 (0)	–
Total tricuspid valve surgery	21/25 (84)		
Tricuspid valve repair	19/23 (83)	0 (0)	0 (0)
Biological prosthesis	2/2 (100)	0 (0)	–
Total aortic valve surgery	17/18 (94)		
Aortic valve repair	2/2 (100)	0 (0)	0 (0)
Mechanical prosthesis	15/16 (94)	0 (0)	–

No patients had moderate or severe paravalvular leakage. Echocardiograms from 2016 were missing; thus, the number of patients is somewhat lower than the number of available patients. n/a (%), number/available (%).

Three surgical patients died within the first 30 days. One died due to refractory severe pulmonary hypertension; the second died due to in-hospital cardiac arrest, with a confirmed low serum potassium level; and the third died after re-hospitalization 23 days after surgery with diarrhea and septic shock.

**Figure 3:**
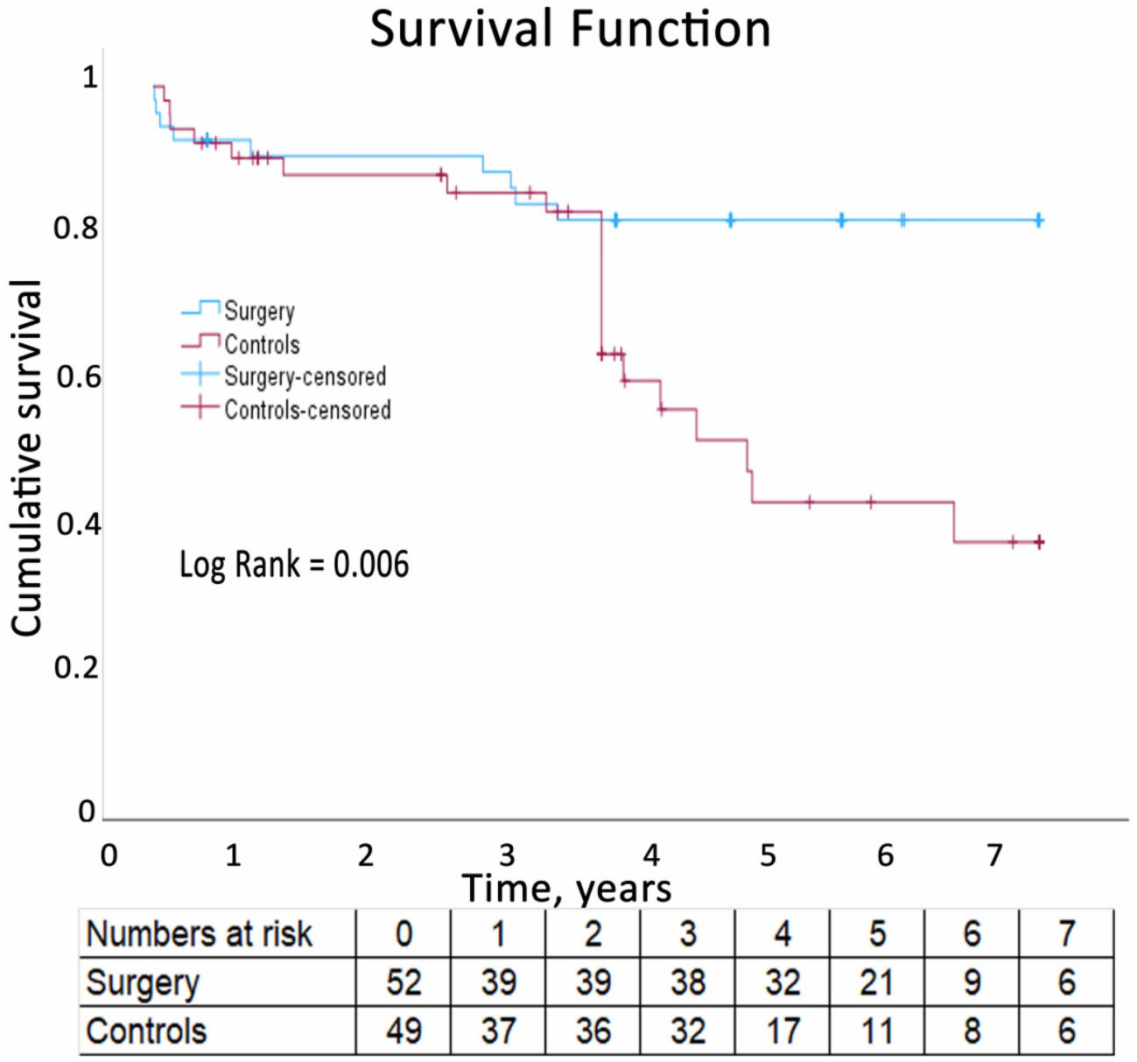
Survival of the operated patients compared to the controls.

Additionally, six patients died after the first month. Thus, there was a total of nine (17%) deaths during the mean (SD) follow-up period of 4.1 (2.5) years. Of the latter six deaths, three died suddenly 64 days, 10 months, and 3.1 years after surgery, respectively. Furthermore, two patients died of heart failure 2.8 and 3.5 years after surgery, respectively. The final patient died 3.1 years after surgery of cardiogenic shock secondary to recurrent malignant arrhythmia. In total, 21 (43%) controls died during follow-up. The survival rates were 83% in the surgical group and 57% in the control group, with a significant difference between the groups, *P* = 0.004 (Figure 3).

### Valvular outcomes

**Figure 4:**
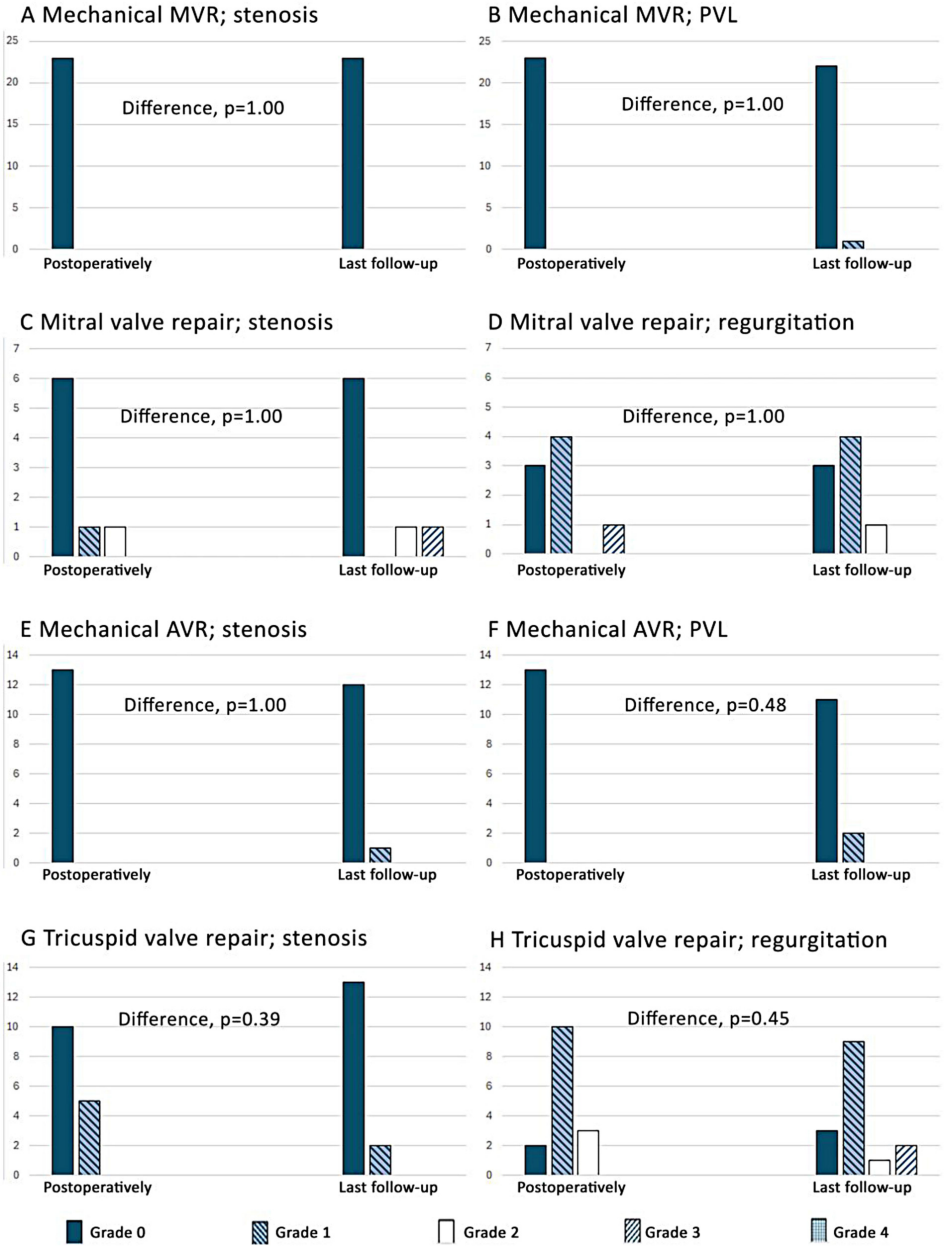
Valvular function postoperatively and at last follow-up of the 43 patients eligible for the mid-term valvular analysis (0 = none, 1 = mild, 2 = mild to moderate, 3 = moderate, and 4 = severe). Trace (0.5) is included in group 1 (mild). Additionally, two patients underwent aortic valve repair, one patient had an mitral valve replacement (MVR) with a biological prosthesis, and one patient had a tricuspid valve replacement (TVR) with a biological prosthesis. All these patients had pathology classified as lower than moderate at their last follow-up. **(A)** Mechanical MVR; stenosis. **(B)** Mechanical MVR; PVL. **(C)** Mitral valve repair; stenosis. **(D)** Mitral valve repair; regurgitation. **(E)** Mechanical AVR; stenosis. **(F)** Mechanical AVR; PVL. **(G)** Tricuspid valve repair; stenosis. **(H)** Tricuspid valve repair; regurgitation.

A total of 43 out of 52 surgical patients, with 71 replaced or repaired valves, were available for analysis of valvular function at their last follow-up. None of the operated valves had more than mild pathology when assessed postoperatively. Figure 4 shows details of the function of the operated valves for the most frequently performed procedures in the patients included in the analyses during follow-up. At the last follow-up, no repaired valves or valvular prostheses had severe dysfunction, none had moderate regurgitation, and only four had moderate obstructions (Table 4). Of the latter, three were valvular repairs (one mitral and two tricuspid) and one was a mechanical mitral prosthesis.

**Table 4:** Data from echocardiographic evaluations at the last follow-up of 43 operated patients.

Variable	*n* (%)	Stenosis/obstruction (≥moderate)	Regurgitation (≥moderate)
Total mitral valve surgery	37 (86)		
Mitral valve repair	8 (100)	1 (12.5)	0 (0)
Mechanical prosthesis	28 (76)	1 (3.6)	–
Biological prosthesis	1 (100)	0 (0)	–
Total tricuspid valve surgery	18 (72)		
Tricuspid valve repair	17 (74)	2 (11.8)	0 (0)
Biological prosthesis	1 (50)	0 (0)	–
Total aortic valve surgery	16 (89)		
Aortic valve repair	2 (100)	0 (0)	0 (0)
Mechanical prosthesis	14 (88)	0 (0)	–

No patients had moderate or severe paravalvular leakage. *n* (%) indicates the number of patients available at the last follow-up related to the total number of operated valves.

### Reproducibility

Supplementary Tables S1 and S2 show the reproducibility of the echocardiographic measurements. Valvular dysfunction grading showed good correlations between echocardiographers (Spearman's *r* 0.74–1) for all valvular pathologies, except for tricuspid regurgitation (Spearman's *r* 0.53), due to disagreements regarding five patients. The good agreement between echocardiographers was further supported by a coefficient of variation ≤10% for the majority of the measurements; however, it was significantly higher for tricuspid valve peak early inflow velocity (21%) and indexed left atrial end-systolic volume (15.5%).

## Discussion

This study presents new data on mid-term outcomes in patients who underwent open-heart surgery for severe chronic RHD in LMICs compared to conservatively treated patients. Survival was better in the surgically treated group than in the controls with similar valve severity characteristics who were not offered surgery due to the limited resources available in this LMIC. The sustained good function of the repaired valves and valvular prostheses throughout the 4-year follow-up period supports that successfully operated patients with severe RHD are expected to do better in the long term than conservatively treated patients (20, 21). With a well-structured cardiac surgery service program, open-heart surgery for severe chronic RHD may also improve patient outcomes in LMICs, even though a number of factors may negatively affect long-term outcomes.

### Population

The characteristics of the surgical group and the selected control candidates were balanced with respect to the severity of valvular disease, age, sex, and time on waiting list as of November 2019. However, of the 200 controls, 43 were not included as they could not be identified. There were differences between the groups with respect to the proportion lost to follow-up, which was significantly higher among the controls. To ensure we did not include a control group with more severe disease than those who were operated on, we chose to start follow-up at the time of being placed on the waiting list for the controls instead of the date of surgery, as for those who were operated on. Thus, it was expected that the surgical group would be older and have more advanced disease and a higher burden of comorbidities than the controls.

At baseline, the surgical patients had more frequent atrial fibrillation, required more anticoagulation therapy, and experienced more thromboembolic events than the controls. Their longer duration since the start of symptoms, older age, and more comprehensive assessment may have influenced this finding. The study population was predominantly young women with long-standing and severe RHD symptoms. Furthermore, congestive heart failure was more common than in a previous publication (3). Even though LV ejection fraction was preserved in the majority of the surgical patients, the low indexed stroke volume of 31 mL/m^2^ and significant pulmonary hypertension indicate more long-standing and advanced RHD.

### Outcomes

The observational design and inherent differences between the groups highlight the fact that controls reflect the natural course of the disease itself. Thus, care should be taken when comparing groups. However, we believe this study provides valuable information due to the lack of existing studies on this topic. Mid-term survival among surgical patients was superior to the natural course of symptomatic patients with RHD with similar characteristics. Some of the postoperative deaths may have been attributed to the patients’ living conditions and access to healthcare facilities. Thus, these could have been avoided in societies with optimal medical follow-up programs.

The presented survival rate after cardiac surgery in this LMIC is comparable to previous studies (17–21). Using follow-up data from all but two surgical patients at a mean of 4.1 years, we found a mortality rate of 17%. In a smaller study of 240 patients with RHD, in the context of 22 international humanitarian missions in Ethiopia, the mortality rate was 7% with a median follow-up of 2 years (17). However, in the latter study, survival data were available from just 69% of the patients at 1 year and 33% at 3 years of follow-up. In the large REMEDY registry of patients with RHD across 14 low- and middle-income countries in Africa and Asia, the mortality rate at 2 years was 17% with 11.5% lost to follow-up (3). Importantly, the REMEDY trial showed a mortality rate of 116/1,000 patient-years in the first year compared to 65/1,000 patient-years in year 2. However, in many reports, large proportions of patients were lost to follow-up, which may have led to an underestimation of mortality (3, 17–21). In our study, only two surgical patients were lost to follow-up and drop-out was low compared to other studies (3, 17–21). Furthermore, outcomes in the control group were in line with previous publications (3, 22–25). However, 94 (60%) of the eligible controls were lost to follow-up and excluded from the analyses, as we were unable to validate information through repetitive phone calls and by scrutinizing medical record files. This influenced the results, likely leading to an underestimation of the mortality and reducing the validity of the clinical outcomes in the control group.

Following the implantation of mechanical valve prostheses, complications related to pregnancy and anticoagulation therapy are important concerns (36, 37). Two female patients, one with a mitral valve repair and one with a mechanical mitral valve, became pregnant and delivered without complications. Two patients were diagnosed with transient dysfunction of their mechanical mitral valves during follow-up. Both were admitted for intensified anticoagulation treatment and recovered. Three patients experienced thromboembolic strokes, of whom one was restricted from receiving warfarin due to the war in the Tigray region, one could not take medicines orally due to typhoid fever and was not supplied with low-molecular-weight heparin, and the last occurred due to poor INR control.

As anticoagulation therapy in resource-scarce environments may be challenging, an INR clinic was established and organized by a local pharmacist, supported by local physicians, to optimize the therapy for the surgical patients. We anticipate that this effort contributed to improved INR monitoring and enhanced the patients' awareness of this critical issue. Moreover, one patient from a rural district performed INR self-monitoring. Furthermore, bioprostheses in such a young population may be associated with poor outcomes (17). Thus, mechanical prostheses were chosen in the majority of patients due to their young age and severe burden of disease and the adequate possibility of monitoring INR. Female patients who wanted to become pregnant were offered valvular repair if possible, but overall, just 8 (21%) of 39 mitral surgeries and 2 (12%) of 17 aortic surgeries were repairs. In contrast, the majority of the tricuspid surgeries were repairs, with bioprostheses used in just 2/21 (10%). Importantly, all the operated valves were patent and well-functioning at the patients’ last follow-up visits. This is encouraging, since concerns related to whether valve replacement with a mechanical prosthesis or valvular repair should be preferred in LMICs have to balance between a dysfunctional prosthesis with thrombotic events due to poor INR control or time-sensitive failure of repaired valves (16, 18, 19, 21).

### Ethical considerations

Since a randomized controlled study would not be ethically acceptable, we constructed a historical control group to provide information regarding the natural history of advanced RHD in this LMIC setting, as cardiac surgery services were unavailable to this subpopulation due to limited availability. Patients with the most severe disease, but still recommended for surgical treatment by the heart team, were prioritized for surgery. Similarly, some patients who were technically suitable were declined for surgery due to high surgical risk (severe heart failure, critical pulmonary hypertension, or severe sequelae following previous stroke) that required perioperative management currently unavailable in LMICs. Importantly, the patients who were declined for surgery were not included as controls. Similarly, controls treated by percutaneous transcatheter mitral valvotomy or surgery performed by other missions were excluded.

### Strength and limitations

The main limitation of this study is the relatively low number of patients included, with 52 patients with RHD who underwent surgery and 49 historical controls. The main strength is the comprehensive pre- and post-operative assessments and complete follow-up data after the surgery. Compared with other studies, we also presented comprehensive echocardiographic data on valve durability and data from a historical control group. Importantly, only two surgical patients were lost to follow-up. Such a low proportion of patients lost to follow-up is rarely the case in similar studies (17–19, 21). It is important to interpret the differences between the groups with caution. As they are not equal, the generalizability of the differences between the groups is limited. Even though we made significant efforts to balance the groups when selecting the controls, we did not randomize patients into the intervention and control groups. Furthermore, a significant difference in data collection between the groups was that no control patient was examined by the Norwegian heart team. Thus, we lack comprehensive information on comorbidity, echocardiographic measurements, and some clinical outcomes in the control group, which, together with the difference in loss to follow-up between the groups, probably underestimates the disease burden in the control group. Moreover, the global COVID-19 pandemic negatively influenced the number of surgeries that could be performed and the war in Tigray negatively influenced the treatment and follow-up of surgical patients. This may have worsened the outcome in some of the surgical patients. In sum, this means that the presented results likely underestimate the effect size of valvular surgery in severe symptomatic RHD on clinical outcomes. Nonetheless, our results add to the knowledge base regarding outcomes after valvular surgery in patients with RHD in LMICs.

## Conclusion

Mid-term outcomes in patients after open-heart surgery for severe chronic RHD in a sub-Saharan country were superior compared to a group of matched controls who did not receive surgery due to the limited availability. The function and durability of the repaired and prosthetic valves were good. Thus, we conclude that open-heart surgery for severe chronic RHD in LMICs may improve long-term outcomes when supported by a dedicated cardiac program including comprehensive follow-up to optimize post-operative care. To succeed in building sustainable cardiac surgery services in LMICs, there is a need for collaboration and support from countries with well-established cardiac surgery services.

## Data Availability

The raw data supporting the conclusions of this article will be made available by the authors, without undue reservation.
