# Supplementary material for: Mid-term outcomes after open-heart surgery for severe chronic rheumatic heart disease in a low-income country: an observational study with historical controls
Source: Front Cardiovasc Med. 2026 Mar 11;13:1763889. doi: 10.3389/fcvm.2026.1763889 (PMC13013438; doi:10.3389/fcvm.2026.1763889)
Supplement: Supplementary file 1 [file Datasheet1.docx]

**Supplementary material.**

**Methods reproducibility.**

Echocardiographic reproducibility analyses were performed by two physician echocardiographers in 15 randomly selected patients using EchoPAC SWO (version 206, GE HealthCare, Horten, Norway). All analyses were performed blinded to each other’s results and the individual patient characteristics excluding the type of surgery being performed.

Valvular function was compared in repaired valves and in valvular prosthesis. Valvular stenoses were graded as: 0, none; 1, mild; 2, mild to moderate; 3, moderate; 4, severe. Valvular and paravalvular regurgitations (prosthetic valves only) were graded as: 0, none; 0.5, trace; 1, mild; 2, mild to moderate; 3, moderate; 4, severe.

Echocardiographic analyses were aligned to current recommendations.

Statistical methods. Categorical data were compared using Spearman’s rank correlation. The corresponding 95% confidence intervals (95% CI) were created using bootstrapping with 100 repetitions. The coefficient of variation was calculated as the within pair of measurements’ standard deviation divided by the mean of each pair and averaged.

**Results – reproducibility.**

**Supplementary table 1.** Spearman rank correlations of categorized valvular function.

| **Variables** | **Number of pairs available** | **r (95% CI)** |
| --- | --- | --- |
| Mitral stenosis | 15 | 0.741 (0.452-1) |
| Mitral regurgitation | 15 | 0.736 (0.208-1) |
| Mitral prosthesis paravalvular leakage | 9 | 1 |
| Aortic stenosis | 15 | 0.997 (0.983-1) |
| Aortic regurgitation | 15 | 1 |
| Aortic prosthesis paravalvular leakage | 3 | 1 |
| Tricuspid stenosis | 9 | 1 |
| Tricuspid regurgitation | 10 | 0.534 (0.401-0.920) |
| Tricuspid prosthesis paravalvular leakage | 0 | - |
| Pulmonary stenosis | 15 | - |
| Pulmonary regurgitation | 15 | 0.817 (0.539-1) |
| Pulmonary prosthesis paravalvular leakage | 0 | - |

**Supplementary table 2.** Coefficients of variation for selected echocardiographic measurements.

| **Variables** | **Numbers of pairs available** | **Coefficients of variation** |
| --- | --- | --- |
| Left ventricular internal end-diastolic dimension, mm | 15 | 3.4% |
| Left ventricular outflow tract diameter, mm | 14 | 2.7% |
| Left ventricular end-diastolic volume, mL | 15 | 10.1% |
| Left ventricular ejection fraction, % | 15 | 8.4% |
| Indexed left atrial end-systolic volume, mL | 14 | 15.5 % |
| Tricuspid annular plane systolic excursion, mm | 14 | 10.5 % |
| Left ventricular outflow tract mean gradient, mm Hg | 14 | 10.1 % |
| Left ventricular outflow tract velocity time integral, cm | 13 | 7.4 % |
| Left ventricular outflow tract max velocity, m · s^-1^ | 13 | 5.9 % |
| Aortic valve mean pressure gradient, mm Hg | 15 | 13.2 % |
| Aortic valve velocity time integral, cm | 13 | 11.3 % |
| Mean gradient mitral valve, mm Hg | 14 | 9.7 % |
| Mitral valve peak early inflow velocity, m · s^-1^ | 14 | 3.4 % |
| Mean gradient tricuspid valve, mm Hg | 3 | 6.6 % |
| Tricuspid valve peak early inflow velocity, m · s^-1^ | 2 | 21.0 % |
| Tricuspid regurgitation maximal velocity, m · s^-1^ | 9 | 8.5 % |
| Right ventricular outflow tract peak velocity, m · s^-1^ | 15 | 7.2 % |
| Pulmonic valve maximal velocity, m · s^-1^ | 15 | 5.0 % |
| Maximum diameter vena cava inferior, mm | 9 | 13.2 % |
